# Confounding and regression adjustment in difference‐in‐differences studies

**DOI:** 10.1111/1475-6773.13666

**Published:** 2021-05-12

**Authors:** Bret Zeldow, Laura A. Hatfield

**Affiliations:** ^1^ Department of Mathematics and Statistics Colby College Waterville Maine USA; ^2^ Department of Health Care Policy Harvard Medical School Boston Massachusetts USA

**Keywords:** difference‐in‐differences, matching, parallel trends, regression adjustment, time‐varying confounding

## Abstract

**Objective:**

To define confounding bias in difference‐in‐difference studies and compare regression‐ and matching‐based estimators designed to correct bias due to observed confounders.

**Data sources:**

We simulated data from linear models that incorporated different confounding relationships: time‐invariant covariates with a time‐varying effect on the outcome, time‐varying covariates with a constant effect on the outcome, and time‐varying covariates with a time‐varying effect on the outcome. We considered a simple setting that is common in the applied literature: treatment is introduced at a single time point and there is no unobserved treatment effect heterogeneity.

**Study design:**

We compared the bias and root mean squared error of treatment effect estimates from six model specifications, including simple linear regression models and matching techniques.

**Data collection:**

Simulation code is provided for replication.

**Principal findings:**

Confounders in difference‐in‐differences are covariates that change differently over time in the treated and comparison group or have a time‐varying effect on the outcome. When such a confounding variable is measured, appropriately adjusting for this confounder (ie, including the confounder in a regression model that is consistent with the causal model) can provide unbiased estimates with optimal SE. However, when a time‐varying confounder is affected by treatment, recovering an unbiased causal effect using difference‐in‐differences is difficult.

**Conclusions:**

Confounding in difference‐in‐differences is more complicated than in cross‐sectional settings, from which techniques and intuition to address observed confounding cannot be imported wholesale. Instead, analysts should begin by postulating a causal model that relates covariates, both time‐varying and those with time‐varying effects on the outcome, to treatment. This causal model will then guide the specification of an appropriate analytical model (eg, using regression or matching) that can produce unbiased treatment effect estimates. We emphasize the importance of thoughtful incorporation of covariates to address confounding bias in difference‐in‐difference studies.

**What is known on this topic?**

- Difference‐in‐difference studies can estimate causal effects of treatment if strong causal assumptions are met.
- Confounding in difference‐in‐difference arises because covariates evolve over time differently in the treated and control groups or because the effects of covariates on outcomes vary over time.
- Time‐varying confounding can bias estimates from difference‐in‐difference designs by violating the causal assumptions.

**What this study adds?**

- Regression and matching techniques to address confounding by observed covariates must be coherent with the underlying causal model to produce unbiased estimates.
- Postulating a causal model of the evolution of covariates in treated and control groups over time and those covariates' relationships to outcomes over time is a crucial prerequisite for any difference‐in‐differences study.

## 1 INTRODUCTION

Difference‐in‐differences (diff‐in‐diff) studies are frequently used to evaluate new policies and programs. For example, hundreds of studies have estimated the effects of expanded Medicaid eligibility through the Affordable Care Act (ACA) in the United States, and many of these used diff‐in‐diff. Following the Supreme Court ruling on the ACA,^1^ each state chose whether to expand its threshold for Medicaid eligibility, which created groups of treated states and comparison (untreated) states and enabled the application of diff‐in‐diff.^2^ These studies have informed ongoing policy debates about the future of the ACA and state Medicaid waivers.

Diff‐in‐diff relies on strong and unverifiable assumptions. The key assumption for diff‐in‐diff is that the outcomes of the treated and comparison groups would have evolved similarly *in the absence of treatment*. Unlike cross‐sectional studies, diff‐in‐diff does not require the treated and comparison groups to be balanced on covariates. Thus, a covariate that differs by treatment group and is associated with the outcome is not necessarily a confounder in diff‐in‐diff. Only covariates that differ by treatment group and are associated with outcome *trends* are confounders in diff‐in‐diff.

In applied literature, many diff‐in‐diff studies are run on autopilot: plot the data, test for parallel trends before the intervention, and fit a regression model that includes an interaction between time and treatment, perhaps with some adjustment for covariates. Rarely are the mechanisms of confounding considered. In this paper, we discuss how diff‐in‐diff requires a different understanding of confounding and regression adjustment than other study designs. We show how covariates, both time‐invariant and time‐varying, affect the causal assumptions and inform analysis choices. Using simulations, we demonstrate how to adjust for confounders using regression and matching. We focus on common diff‐in‐diff models with a single start date for a binary treatment and no unobserved treatment effect heterogeneity. To applied researchers, we offer strategies to estimate unbiased causal effects by combining subject matter expertise with thoughtful modeling.

### 1.1 Parallel trends

In cross‐sectional studies, the definition of a confounder comes from the assumption that potential outcomes are independent of treatment. Colloquially, we say that a confounder is a covariate related to both treatment and outcome, and we must condition on all confounders to ensure independence between treatment and potential outcomes. VanderWeele and Shpitser noted the lack of rigor in the definition of a confounder.^3^ In this spirit, we examine confounding in diff‐in‐diff.

First, we define time‐varying and time‐invariant covariates and time‐varying effects of covariates. A time‐varying covariate is one that changes over time for a unit, whereas a time‐invariant covariate does not change over time for a unit. For example, a person's weight is time‐varying while their place of birth is time‐invariant. A covariate that has a time‐varying effect on an outcome is different than the (in)variance of the covariate itself. When a covariate affects the outcome differently over time, we say it has a time‐varying effect on the outcome.

In diff‐in‐diff, our target estimand is the average effect of treatment on the treated (ATT),

(1) $$\mathrm{ATT}\left(t^{*}\right)=E\left\{Y^{1}\left(t^{*}\right)-Y^{0}\left(t^{*}\right)|D=1\right\},$$

for some time *t* ^*^ ≥ *T*_0_ after the intervention is introduced to the treatment group (*T*_0_). In this expression, *D* = 1 indicates the treated group and *Y* ^*d*^(*t*) is the potential outcome at time *t* under treatment *d*. Note that Equation (1) contains the posttreatment untreated outcome in the treated group, *Y* ^0^(*t* ^*^), which we can never observe. However, with some additional assumptions, we can re‐write the target estimand in a form that contains only observables, a process known as identification. Below, we describe assumptions that allow us to identify the ATT.

First, we assume no anticipation effects, that is, potential outcomes are not affected by future treatment. From this, it follows that the observed and potential outcomes are the same at pretreatment times, *Y*(*t*) = *Y* ^0^(*t*) = *Y* ^1^(*t*) for *t* < *T*_0_. Second, we assume that we can observe the potential outcomes corresponding to actual treatment received, *Y*(*t*) = *Y* ^0^(*t*)(1 − *D*) + *Y* ^1^(*t*)*D*.

Third, we make the so‐called “parallel trends” assumption, which we define first in the simple setting of one pretreatment time (*t* = 0) and one posttreatment time (*t* = 1):

(2) $$E\left\{Y^{0}\left(1\right)-Y^{0}\left(0\right)|D=0\right\}=E\left\{Y^{0}\left(1\right)-Y^{0}\left(0\right)|D=1\right\}.$$

Under parallel trends we assume the change in the average untreated potential outcomes from pre‐ to posttreatment is the same in the treated and comparison groups. Since the untreated potential outcome in the posttreatment period *Y* ^0^(1) is not observable in the treated group, this assumption is untestable.

This definition of parallel trends with two time points is nearly universal in the diff‐in‐diff literature.^4^ However, many applications consider more than two time points, so we extend the assumption accordingly. In the strictest version of parallel trends, every pair of time points satisfies Equation (2). That is,

(3) $$E\left\{Y^{0}\left(t^{*}\right)-Y^{0}\left(t^{\prime }\right)|D=0\right\}=E\left\{Y^{0}\left(t^{*}\right)-Y^{0}\left(t^{\prime }\right)|D=1\right\},$$

for *t* ^*^ ≠ *t*′. While we can relax this, many researchers have this version in mind when testing for parallel trends in the preintervention periods, contending that evidence of parallel trends before treatment strengthens the plausibility of parallel trends over the whole study period.^5^

Given these assumptions, we can now rewrite the ATT in a form involving only observable quantities^6^:

$$\mathrm{ATT}\left(t^{*}\right)=\left[E\{Y\left(t^{*}\right)|D=1\}-E\{Y\left(t^{\prime }\right)|D=1\}\right]-\left[E\{Y\left(t^{*}\right)|D=0\}-E\{Y\left(t^{\prime }\right)|D=0\}\right],$$

with *t* ^′^ < *T*_0_ ≤ *t* ^*^. To estimate this quantity, we can select from a variety of techniques, ranging from simple nonparametric estimators based on sample means to more sophisticated regression models.

We start by specifying a model for the untreated potential outcomes. Following convention in diff‐in‐diff literature,^7^ we write the untreated potential outcome of the *i*th unit as

(4) $$E\left[Y_{i}^{0}\left(t\right)|D=d,X=x_{\mathrm{it}}\right]=\alpha_{0}+\alpha_{1}d_{i}+\zeta_{t}+\lambda_{t}x_{\mathrm{it}},$$

where *ζ*_*t*_ are time fixed effects, *d*_*i*_ is an indicator for the treated group, and *x*_*it*_ is a covariate that can vary across units *i* and time *t*. The coefficients are an intercept, *α*_0_; a constant difference between treated and comparison groups, *α*_1_; and the effect of the covariate on the outcome at time *t*, *λ*_*t*_.

So far, we have only considered untreated potential outcomes. Next, we write the data‐generating model for the treated potential outcomes by assuming a constant, additive effect of treatment,

$$Y_{i}^{1}\left(t\right)=Y_{i}^{0}\left(t\right)+\gamma ,$$

which implies

$$E\left[Y_{i}^{1}\left(t\right)|D=1,X=x_{\mathrm{it}}\right]=\alpha_{0}+\alpha_{1}d_{i}+\zeta_{t}+\lambda_{t}x_{\mathrm{it}}+\gamma .$$

With these data‐generating models, we can establish conditions in which the covariate can confound the treatment effect *γ*. (Proofs of these are found in Appendix A in Supporting Information.)

First, consider a time‐invariant covariate. Parallel trends hold if either: (1) the means of the covariate are the same in both treated and comparison groups or (2) the effect of the covariate on the outcome is the same across time. Thus, a time‐invariant covariate is a confounder if the means of the covariate are different in the two groups *and* it has a time‐varying effect on the outcome.

Next, consider a time‐varying covariate. Parallel trends hold if: (1) the means of the covariate are the same in both treated and comparison groups (and evolve the same over time) or (2) the relationship of the covariate to the outcome is constant *and* the difference in the mean of the covariate between groups is constant over time. Thus, a time‐varying covariate is a confounder if (1) the covariate means evolve differently between the two groups *or* (2) the covariate means start at different levels and evolve in parallel, and the covariate has a time‐varying effect on the outcome.

Putting this all together, a confounder in diff‐in‐diff is a variable with a time‐varying effect on the outcome or a time‐varying difference between groups. The parallel trends assumption ensures that group‐invariant time trends or time‐invariant level differences between the groups are not problematic. However, time‐varying differences between groups, due to covariates with an evolving relationship to the outcome or differential evolution in the groups, can cause confounding bias.

Compare this to the definition of a confounder in cross‐sectional settings, which is a variable associated with both treatment and outcome. In diff‐in‐diff, a confounder always has some time‐varying effect: either the relationship of the variable to the outcome changes over time or the variable evolves differently between the groups over time.

Next, we consider adjusting for these types of confounding variables. An effective adjustment strategy must account for the covariate's time‐varying differences between groups or its time‐varying effect on the outcome. In addition to regression adjustment, we also consider matching^8^, ^9^ in the section titled “What about Matching?”

### 1.2 Adjusting for confounders

We use a linear regression model to estimate the ATT *γ* in the presence of a confounder *X*. In our simulations, we explore models of the following form:

$$E\left[Y_{i}\left(t\right)|D=d_{i},X=x_{\mathrm{it}}\right]=\alpha_{0}+\zeta_{t}+\alpha_{1}d_{i}+\lambda_{t}x_{\mathrm{it}}+\gamma p_{t}d_{i},$$

where ζ_*t*_ are time fixed effects, α_1_ is the constant difference between treated and comparison groups, and *p*_*t*_ is an indicator for posttreatment time points. The coefficient γ on the interaction between treatment group and postintervention times, *p*_*t*_ *d*_*i*_, is the ATT when the model is correctly specified.

The correct form for regression models that account for confounding depends on whether the covariate is time‐invariant or time‐varying and whether its effect on the outcome is constant or time‐varying. We consider models that include constant (main) effects of time‐invariant and time‐varying covariates (*λx*_*i*_ and *λx*_*it*_) or time‐varying (interactions with time) effects of covariates (*λ*_*t*_ *x*_*i*_ and *λ*_*t*_ *x*_*it*_).

#### 1.2.1 Adjusting for time‐invariant confounders

When *X* is a time‐invariant confounder, linear regression with a (time‐invariant) main effect will not eliminate bias. Nevertheless, practitioners often adjust for main effects only,^10^, ^11^, ^12^, ^13^ perhaps out of habit. A simple demonstration will show that adjusting only for main effects is ineffective in correcting nonparallel trends. Suppose we have a time‐invariant covariate *x*_*i*_ with different means in the two groups at baseline, *E*[*X*∣ *D* = 0] ≠ *E*[*X*∣ *D* = 1] . To be a confounder, it must have a time‐varying effect. Recall that confounding arises because of a covariate's effect on parallel trends, which involve only the untreated outcomes, so we ignore treatment effects. Thus, the treated and untreated potential outcomes are the same, and we can illustrate our points in observed data. Outcomes are generated from Equation (4) with a time‐varying relationship between the covariate and outcome and different covariate means in the treated and comparison groups.

**FIGURE 1:**
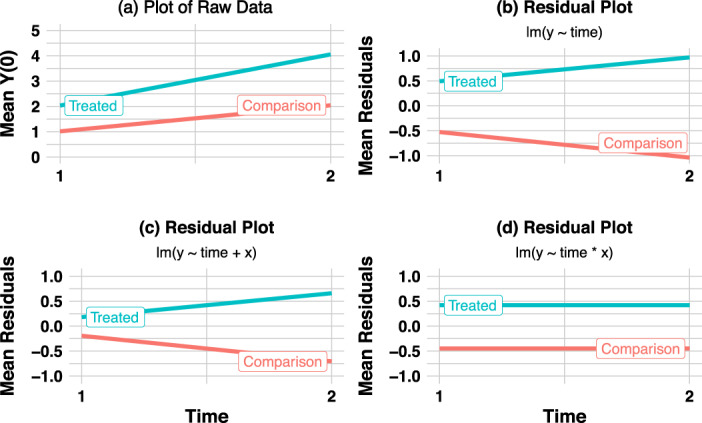
Adjusting for the main effect of a covariate does not correct for diverging trends, but adjusting for its interaction with time does. Legend: In this simulated example, untreated potential outcomes depend on a time‐invariant covariate with a time‐varying effect. Panel A shows mean untreated potential outcomes by group. Panels B to D show residuals from linear models, denoted using pseudo‐code for the function lm, which fits a linear model for outcome y. In panel B, the only predictor is time. In panel C, the predictors are time and the covariate x. In panel D, the predictors are time, the covariate, and their interaction [Color figure can be viewed at wileyonlinelibrary.com]

In Panel A of Figure 1, we plot the mean outcomes by group and time, and the nonparallel outcome evolution is apparent. Panel B shows residuals from a simple linear regression with only a time effect. In Panel C, we add a main effect for the covariate *X* to the model. However, Panels B and C still show diverging trends. In Panel D, we add an interaction between *X* and time. Only in Panel D do we properly account for the time‐varying nature of the confounder and obtain an unbiased result (recall the true treatment effect is zero here).

#### 1.2.2 Adjusting for time‐varying confounders

Time‐varying confounders can also invalidate parallel trends and introduce bias into our estimate of the ATT. If we adjust for time‐varying confounders by including the main effect or its interaction with time in a regression, we risk conditioning on posttreatment covariates that may be affected by treatment. As Rosenbaum notes, at best, adjusting for posttreatment covariates provides no benefit; at worst, it may introduce additional bias.^14^ This occurs because the time‐varying covariate can act as both a confounder and a mediator. As such, when trying to recover the ATT via regression, the usual interaction parameter may not be an unbiased estimate of the ATT.

Imagine three scenarios: (a) the time‐varying covariate changes in a way completely unrelated to treatment, (b) the time‐varying covariate changes in a way wholly determined by treatment, and (c) the time‐varying covariate changes in a way determined by a combination of treatment and other factors. Whenever (b) or (c) is true and the time‐varying covariate is a cause of the outcome, the ATT is a combination of the direct effect of treatment and the indirect effect of treatment via the covariate. As a result, the regression parameter on the interaction between treatment and the posttreatment indicator may not equal the ATT, even adjusted for the time‐varying covariate. However, if we fail to account for the covariate, we face parallel trends violations. For more details, see in Supporting Information.

#### 1.2.3 What about matching?

##### Matching on time‐invariant covariates

Through matching, we aim to reduce confounding bias by selecting units from the treated and comparison groups that have similar observable characteristics, eliminating imbalances between the groups — a key ingredient in confounding. When matching, we can match observations on pretreatment outcomes, pretreatment covariates, or some combination.

Matching on pretreatment outcomes allows us to use an alternative assumption to estimate the causal effect. This assumption — independence between potential outcomes and treatment assignment conditional on past outcomes — is the basis of lagged dependent variables regression and synthetic control methods.^6^, ^7^, ^15^ However, matching on pretreatment outcomes in diff‐in‐diff can yield unwanted results. In some settings, it reduces bias,^8^, ^9^ while in others, matching induces regression to the mean and *creates* bias.^7^, ^16^

##### Matching on time‐varying covariates

Matching only on time‐invariant pretreatment covariates is attractive because it removes covariate differences between groups. Matching on time‐varying covariates in the pretreatment period can produce bias due to regression to the mean. Moreover, if confounding arises because of differential evolution of the covariate in the two groups, matching only on pretreatment values will be insufficient to address the confounding. While it may be tempting in this case to match on both pre‐ and posttreatment values of a time‐varying covariate, matching on posttreatment variables that may be affected by treatment can produce causal estimates that do not equal the ATT.^14^ For this reason, we do not explore strategies that match on posttreatment covariates. Clearly, choosing the right matching variables is the key to effective matching. A good overview on the current state of matching for diff‐in‐diff is provided by Lindner and McConnell.^17^

Returning to the demonstration of parallel trends in Figure 1, matching on the pretreatment covariate also fixes diverging trends. Eliminating the difference between the covariate means in the treated and comparison group via matching is sufficient to address confounding. If the confounding had arisen due to a time‐varying covariate, the strategy would not suffice.

## 2 METHODS

As we have discussed, both matching and regression adjustment have limitations. We conduct simulation studies to illustrate the advantages and shortcomings of regression and matching techniques that are commonly employed by practitioners of diff‐in‐diff. In each simulation scenario, we generate 400 datasets of *n* = 800 units observed at *T* = 10 time points. The first five time points are pretreatment times, and the rest are posttreatment. Each unit is assigned to the treatment group with probability 0.5. To each simulated data set, we apply regression and matching techniques and compare the bias of the resulting treatment effect estimates.

We simulate data and analyze it using the R environment.^18^ We fit regression models using the lm function and estimate post hoc, cluster‐robust SEs using the cluster.vcov function in the multiwayvcov package.^19^ For our matching estimators, we implement nearest neighbor matching with replacement using the MatchIt package.^20^ We present averages, across simulated data sets, of the absolute percent bias and SE of the estimated treatment effects. Mean absolute percent bias is calculated by taking the average of all estimates, subtracting the true value of the ATT, taking the absolute value, and converting it to a percentage relative to the true ATT. Mean SE is the mean of the 400 SE estimates.

Below, we describe the specifics of our data‐generating and analysis models, first for scenarios with time‐invariant covariates and then for scenarios with time‐varying covariates. Table 1 gives an overview of the data‐generating process for each simulation scenario; more detail is provided in Table D1 in Supporting Information. Simulation code is on GitHub (https://www.github.com/zeldow/DID-confounding-supplementary).

**TABLE 1:** Illustration of the data‐generating processes for simulation studies [Color table can be viewed at wileyonlinelibrary.com]

Simulation scenario	Covariate evolution by group	Covariate effect over time	Confounded?
1: Time‐invariant covariate effect	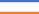 Constant	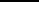 Constant	No
2: Time‐varying covariate effect	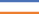 Constant	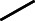 Varying	Yes
3: Treatment‐independent covariate effect	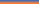 Equal	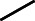 Varying	No
4a: Parallel evolution	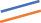 Parallel	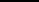 Constant	No
5a: Evolution differs by group	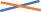 Divergent	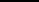 Constant	Yes
6a: Evolution diverges in postperiod	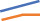 Diverges in post	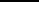 Constant	Yes
4b: Parallel evolution	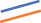 Parallel	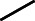 Varying	Yes
5b: Evolution differs by group	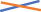 Divergent	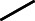 Varying	Yes
6b: Evolution diverges in postperiod	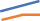 Diverges in post	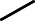 Varying	Yes

*Note*: The column showing covariate evolution by group shows how the simulated covariate differs by treatment group, indicated by the two different lines. The covariate effect over time column shows how the covariate affects the outcome over time (ie, is there an interaction between the covariate and time?). The last column tells us whether or not the scenario has confounding. For scenarios without confounding, a simple unadjusted difference‐in‐differences estimator will recover the true treatment effect.

### 2.1 Time‐invariant covariate

#### 2.1.1 Data‐generating models

Our first set of simulations involves a time‐invariant covariate. In Scenario 1, the distribution of *X* differs by treatment group, but *X* has a time‐invariant effect on the outcome *Y*. Scenario 2 is the same as Scenario 1 but we allow the effect of *X* on *Y* to be time‐varying. In Scenario 3, the effect of *X* on *Y* is again time‐varying, but the distribution of *X* is the same in the treated and control groups.

In Scenarios 1 and 3, analyses that do not adjust for *X* will be unbiased, because *X* does not satisfy the definition of a confounder. In Scenario 1, this is because *X* does not have a time‐varying effect on *Y*; in Scenario 3, this is because the distribution of *X* is the same in both groups. In Scenario 2, only analyses that adjust appropriately for the time‐varying effect of *X* on *Y* will yield unbiased results. For all three scenarios, the ATT equals the regression parameter which was set to 1. We measure bias with respect to this true ATT.

#### 2.1.2 Analysis approaches

We use both matched and unmatched regression to analyze the simulated data. All regression models include time fixed effects and indicators for treatment, the postperiod, and their interaction. The simple model includes only those elements, ignoring the covariate entirely:

(5) $$E\left(y_{\mathrm{it}}\right)=\alpha_{0}+\zeta_{t}+\alpha_{1}d_{i}+\gamma p_{t}d_{i}.$$

The covariate adjusted (CA) model adjusts for the covariate with a constant coefficient:

(6) $$E\left(y_{\mathrm{it}}\right)=\alpha_{0}+\zeta_{t}+\alpha_{1}d_{i}+\lambda x_{\mathrm{it}}+\gamma p_{t}d_{i}.$$

The time‐varying adjusted (TVA) model allows the coefficient on the covariate to vary over time:

(7) $$E\left(y_{\mathrm{it}}\right)=\alpha_{0}+\zeta_{t}+\alpha_{1}d_{i}+\lambda_{t}x_{\mathrm{it}}+\gamma p_{t}d_{i}.$$

**FIGURE 2:**
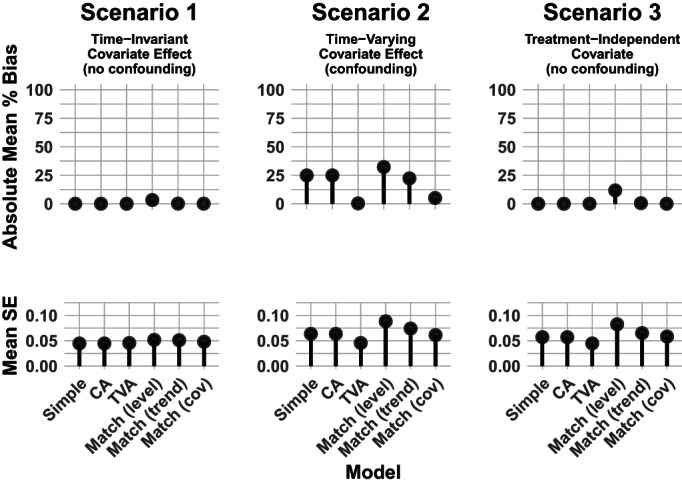
Simulation results for a time‐invariant covariate. Legend: Six regression and matching methods were compared across three simulation scenarios. Each panel shows results from 400 simulated datasets of 800 units each. In Scenario 1, the distribution of the covariate varied by treatment group but the covariate's effect on the outcome did not change (ie, no interaction between the covariate and time). In Scenario 2, the covariate's effect on the outcome changed over time. In the third scenario, the distribution of the covariate was the same in the treated and comparison groups, and the covariate's effect on the outcome changed over time. All analyses were assessed on the mean percent bias and mean standard error (SE) of the effect estimate. CA = Covariate‐adjusted; TVA = Time‐varying adjusted

**FIGURE 3:**
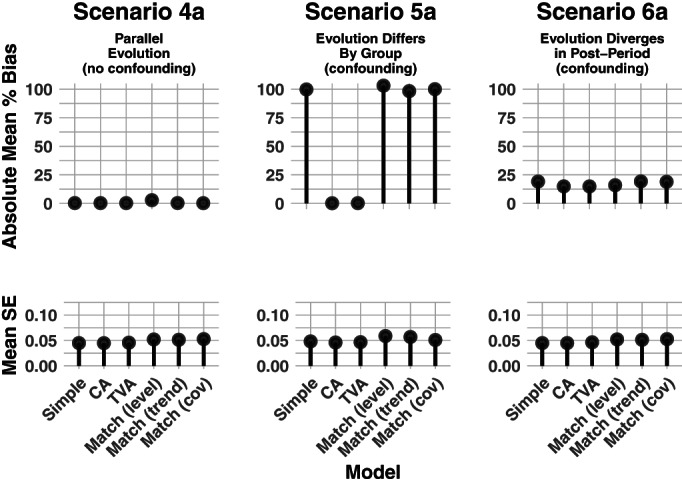
Simulation results for a time‐varying covariate with a time‐invariant effect on the outcome. Legend: Six regression and matching methods were compared across three simulation scenarios. Each panel shows results from 400 simulated datasets of 800 units each. For all scenarios, the covariate's effect on the outcome was constant over time. In Scenario 4a, the time‐varying covariate evolved in the same way for the treated and comparison group. In Scenario 5a, the covariate evolved differently between the two groups starting from the first timepoint (before treatment was implemented). In Scenario 6a, the covariate evolved the same prior to treatment. Once treatment was implemented, evolution of the covariate diverged relative to the two groups. All analyses were assessed on the mean percent bias and mean standard error (SE) of the effect estimate. CA = Covariate adjusted; TVA = Time‐varying adjusted

**FIGURE 4:**
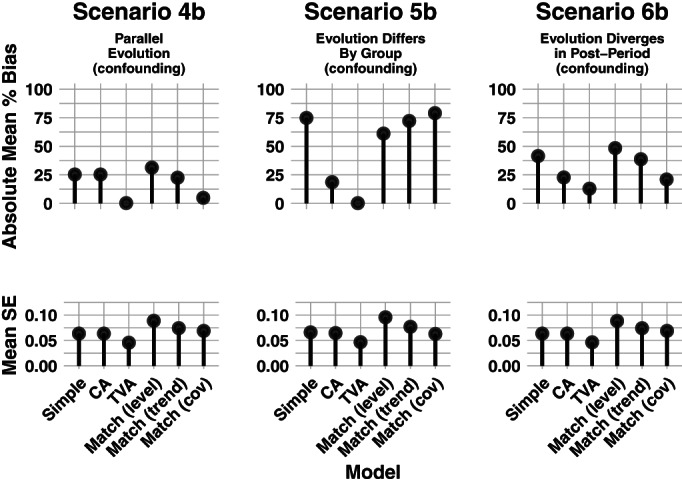
Simulation results for a time‐varying covariate with a time‐varying effect on the outcome. Legend: Six regression and matching methods were compared across three simulation scenarios. Each panel shows results from 400 simulated datasets of 800 units each. For all scenarios, the covariate's effect on the outcome differed across time. In Scenario 4b, the time‐varying covariate evolved in the same way for the treated and comparison group. In Scenario 5b, the covariate evolved differently between the two groups starting from the first timepoint (before treatment was implemented). In Scenario 6b, the covariate evolved the same prior to treatment. Once treatment was implemented, evolution of the covariate diverged relative to the two groups. All analyses were assessed on the mean percent bias and mean standard error (SE) of the effect estimate. CA = Covariate adjusted; TVA = Time‐varying adjusted

Our matching strategies include matching on both outcomes and covariates. We use nearest‐neighbor matching to create three matched data sets, to which we fit the model in Equation (5). The first is matched on the vector of pretreatment outcomes [denoted “match (level)” in Figures 2, 3, 4], the second on the vector of pretreatment outcome first differences [denoted “match (trend)”], and the third on pretreatment covariates [denoted “match (cov)”]. Once we had a matched dataset, we fit the regression model in Equation (5).

### 2.2 Time‐varying covariate

#### 2.2.1 Data‐generating models

The second set of simulations involves a time‐varying covariate, which may evolve differently in the treated and comparison groups. The setup of these simulations is the same as in Scenarios 1 through 3. We include three types of covariate evolution. In Scenario 4, the covariate evolves the same for both the treated group and the comparison group; in Scenario 5, the covariate evolves differently starting from baseline; and in Scenario 6, the covariate evolves the same in the two groups before treatment but differently after treatment.

For all these scenarios, we have two outcome processes: (a) the covariate has a time‐invariant effect on the outcome and (b) the covariate has a time‐varying effect on the outcome. The data‐generating distributions are summarized in Table 1 with more detail in Table D2 of Supporting Information. For scenarios 4 and 5, the ATT equals the regression parameter (set to 1). However, scenario 6 has a covariate that is changed by treatment, acting in part as a mediator. Thus, for scenario 6, the ATTs are 0.85 and 0.87 for outcome processes (a) and (b), respectively. These calculations are provided in Supporting Information.

#### 2.2.2 Analysis approaches

The analysis methods are the same as for time‐invariant covariates (see above), with one exception: in the third matched data set [“match (cov)”], we match on the *vector* of pretreatment covariate values.

## 3 RESULTS

### 3.1 Time‐invariant covariate

Figure 2 shows the results of applying each of the analysis approaches to the data generated with a time‐invariant covariate (Tables 1 and D1). In Scenario 1, while *X* is associated with treatment, it is not a confounder because the effect does not vary over time. Thus, the unadjusted analysis (simple model) is unbiased and adjusting for *X* in the CA and TVA models does not affect either bias or SEs. The results from our matched regressions are similar to those from the unmatched regressions.

In Scenario 2, the time‐varying effect of *X* on *Y* makes *X* a confounder and thus requires covariate adjustment with a time‐varying aspect. Adjusting for the main effect of *X* (CA model) does not alleviate bias or reduce the estimate's SE. Fortunately, we can address the bias by adjusting for the interaction of *X* with time (TVA model). Of the matching strategies, only matching on the covariate effectively eliminates bias.

In Scenario 3, the simple model is already unbiased because *X* is not a confounder. In fact, all estimation strategies yield unbiased estimates except matching on pretreatment outcomes, which is biased by about 10% due to regression to the mean. We see about 20% lower mean SE when we adjust for the covariate in the TVA model compared to the simple model.

### 3.2 Time‐varying covariate

Figures 3 and 4 show the results of applying each of the analysis approaches to the data generated using time‐varying covariate processes (Table 1 and Table D2). In Scenario 4a, there is no confounding when the effect of *X* on *Y* is constant over time, and the mean of *X* evolves the same for each group. As a result, each modeling strategy is unbiased. However, when *X* has a time‐varying effect on *Y* in Scenario 4b, *X* is a confounder and only time‐varying adjustment (TVA) eliminates bias. Matching on the vector of pretreatment values of *X* nearly eliminates the bias.

In Scenario 5, the time‐varying covariate evolves differently by group, beginning at baseline. When the effect of *X* on the outcome is constant (Scenario 5a), we can simply adjust for time‐varying *X* (CA model) to eliminate confounding bias. When the effect of *X* on *Y* varies over time (Scenario 5b), we must adjust for the interaction of *X* and time (TVA model). All the matching strategies have significant bias.

In Scenario 6, the time‐varying covariate evolves differently by group, but only *after* the treatment is introduced at *t* = 6. Recall that in this scenario, the ATT does not simply equal the regression coefficient on an interaction term. Thus, in both Scenarios 6a and 6b, we have significant confounding bias in our estimates and never succeed in recovering the true ATT.

## 4 DISCUSSION

We contribute to diff‐in‐diff literature by examining how observable covariates may violate causal assumptions and comparing regression and matching strategies to adjust for confounders. It is tempting to toss all observed covariates into a regression model, but the form of the model specification should be tailored to address time‐varying confounding. Our findings have several limitations, discussed below.

First, adjusting for confounders may be untenable for sparse data. Regression adjustment depends on knowing and measuring the confounders as well as the functional form of their effects on the outcome. The true relationship between covariates and treatment and outcomes over time may be complex and involve high‐dimensional interactions. All of the usual cautions about parametric regression models apply here.^21^ An alternative doubly robust method has recently been proposed that may avoid some of the pitfalls of correct outcome regression model specification by introducing a second opportunity to ameliorate confounding.^22^

Second, our conclusions only apply to linear models; nonlinear models present different challenges.^23^ For one, the interaction term in generalized linear models is difficult to interpret.^24^ Moreover, it is well‐known limitation of diff‐in‐diff that the parallel trends assumption is scale‐dependent. Even a seemingly innocuous outcome transformation, transforming dollars to log dollars, can have serious implications for the underlying causal assumptions. Two groups satisfying parallel trends on the log dollars scale will not necessarily satisfy parallel trends on the original scale.^6^ Addressing scale‐invariance is beyond the scope of this paper but has been broached elsewhere.^25^

Third, our paper only does not consider heterogeneous treatment effects. As other authors have noted, model specification often imposes additional assumptions on the treatment effect. For example, the unit‐and time‐fixed effects regression requires treatment effects to be homogeneous with respect to the covariates and no divergent outcome evolution across units with different values of the covariate.^22^ “Expected gains bias” is one form of treatment effect heterogeneity that can limit the generalizability of our conclusions.^26^, ^27^ If some units are able to select their treatment group based on its perceived benefit, the treatment effect estimated in the study sample will not match the population ATT. However, expected gains biases are typically driven by unobserved characteristics, but we focus entirely on observed variables.

Lastly, recovering the ATT in diff‐in‐diff can be difficult, especially with time‐varying confounders. Goodman‐Bacon notes that adding time‐varying covariates adds a new source of identifying variation and changes the decomposition of the regression parameter.^28^ None of our analysis methods produced an estimate that equaled the true ATT in the scenario with a time‐varying covariate affected by treatment. In this scenario, the parameter from regression is not an estimate of the ATT and should not be interpreted as such.

Done properly, regression adjustment can alleviate bias caused by diverging trends due to measured confounders. Further, even in the absence of confounding, adjusting for covariates can improve efficiency of the effect estimate (see the SE of Scenario 3 in Figure 2). A correctly specified regression approach avoids conditioning on pretreatment outcomes and thus is not susceptible to regression to the mean as some matching methods are.^16^ Lastly, our regression adjustment strategy is agnostic to the structure of the data, whether we have panel data or repeated cross sections. Our simulations assumed panel data, but our results will hold for repeated cross sections. Matching on repeated cross sections is trickier, since some covariates will necessarily be measured on different subjects at different time points, but it is possible.^29^ Both matching and regression adjustment have clear pitfalls (discussed in the above paragraphs), and both have strengths in diff‐in‐diff applications. Deciding which to implement must be done carefully and depends on various factors, including data structure, which covariates are measured, and how many units are in the dataset. Our goal in this paper is not to provide guidance in choosing between matching and regression adjustment. However, in our simple simulations, matching was not better than regression adjustment, and in some cases, it increased bias. We only implemented nearest neighbor matching with replacement; many other matching techniques are possible.

For applied researchers using diff‐in‐diff, we recommend several steps for addressing confounding. First, researchers should clearly specify a causal model and explain how the inclusion of covariates and their functional forms conforms to their assumptions about the relationships among covariates, treatments, and outcomes over time. This begins by writing out the full model specification and by providing analysis code in Supporting Information. Each covariate and coefficient should correspond to a threat to the validity of parallel trends and provide a remedy. We recommend researchers comprehensively list covariates — both observed and unobserved — that might cause violations of parallel trends. The list should contain information on whether the variable is observed, whether the distribution of the covariate is expected to differ in the treatment and comparison groups, whether the covariate is time‐varying, whether its effect on the outcome is likely to vary over time, and whether the covariate may be causally affected by treatment. Such a list is critical to choosing an analytical approach that is suited to the true underlying data‐generating model. For example, if many unobserved covariates are a concern, the analyst may choose a different estimator (instead of one that relies on diff‐in‐diff and the parallel trends assumption). On the other hand, a single time‐invariant confounder with a simple linear relationship to the outcome suggests a straightforward regression approach. Other authors have given similar advice, stressing attention to the reasons for baseline differences between the treated and comparison groups and how these differences might affect parallel trends.^30^

Being thorough in our diff‐in‐diff studies will strengthen conclusions and help alleviate concerns on the credibility of parallel trends. We expect diff‐in‐diff to continue its critical role in informing policy decisions for the foreseeable future. Further development of diff‐in‐diff methodology should involve cooperation among statisticians, epidemiologists, economists, political scientists, and policy analysts.

## Supporting information


**Appendix** **S1.** Supporting Information.
