# Supplementary material for: Confounding and regression adjustment in difference‐in‐differences studies
Source: Health Serv Res. 2021 May 12;56(5):932–41. doi: 10.1111/1475-6773.13666 (PMC8522571; doi:10.1111/1475-6773.13666)
Supplement: Supplementary file 1 — Appendix S1. Supporting Information. [file HESR-56-932-s001.docx]

Supplemental Materials for “Confounding and Regression Adjustment in Difference-in-Differences Studies”

# Appendix A: Conditions for Satisfying Parallel Trends

We demonstrate the conditions for parallel trends to hold in the data-generating model in Eq. (4) in the simple case of only two time points, $t\in\{0,1\}$. Denote the group-time mean of the covariate $E[X_{it}|D=d]$ by $\tau_{t}^{d}$.

We begin with expressions for the mean change in untreated counterfactual outcomes for each group, by plugging Eq. (4) into the parallel trends assumption of Eq. (2). In the treated group, the change over time is

$$E\left[ Y^{0}\left( 1 \right)-Y^{0}\left( 0 \right) \mid D=1 \right]=\left( \alpha_{0}+\alpha_{1}+\zeta_{1}+\lambda_{1}\tau_{t=1}^{d=1} \right)-\left( \alpha_{0}+\alpha_{1}+\zeta_{0}+\lambda_{0}\tau_{t=0}^{d=1} \right)$$

$$=\zeta_{1}-\zeta_{0}+\lambda_{1}\tau_{t=1}^{d=1}-\lambda_{0}\tau_{t=0}^{d=1}$$

and for the comparison group, it is

$$E\left[ Y^{0}\left( 1 \right)-Y^{0}\left( 0 \right) \mid D=0 \right]=\left( \alpha_{0}+\zeta_{1}+\lambda_{1}\tau_{t=1}^{d=0} \right)-\left( \alpha_{0}+\zeta_{0}+\lambda_{0}\tau_{t=0}^{d=0} \right)$$

$$=\zeta_{1}-\zeta_{0}+\lambda_{1}\tau_{t=1}^{d=0}-\lambda_{0}\tau_{t=0}^{d=0}$$

Subtracting the two, we get the differential change in untreated counterfactual outcomes between treated and comparison groups:

$\left( \zeta_{1}-\zeta_{0}+\lambda_{1}\tau_{t=1}^{d=1}-\lambda_{0}\tau_{t=0}^{d=1} \right)-\left( \zeta_{1}-\zeta_{0}+\lambda_{1}\tau_{t=1}^{d=0}-\lambda_{0}\tau_{t=0}^{d=0} \right)$

$$\begin{aligned} =\lambda_{1}\left( \tau_{t=1}^{d=1}-\tau_{t=1}^{d=0} \right)-\lambda_{0}\left( \tau_{t=0}^{d=1}-\tau_{t=0}^{d=0} \right)\#\left( 8 \right) \end{aligned}$$

The parallel trends assumption in Eq. (2) constrains this difference to be 0. Given the data-generating model in Eq. (4), we can put conditions on the $\lambda$’s and $\tau$’s that will ensure parallel trends holds.

For a time-invariant covariate, we write the mean of $X$ in the treated group $\tau_{t=0}^{d=1}=\tau_{t=1}^{d=1}=\tau^{d=1}$ and in the comparison group as $\tau_{t=0}^{d=0}=\tau_{t=1}^{d=0}=\tau^{d=0}$. The differential change in Eq. (8) simplifies to

$$\begin{aligned} \lambda_{1}\left( \tau^{d=1}-\tau^{d=0} \right)-\lambda_{0}\left( \tau^{d=1}-\tau^{d=0} \right)=\left( \lambda_{1}-\lambda_{0} \right)\left( \tau^{d=1}-\tau^{d=0} \right)\#\left( 9 \right) \end{aligned}$$

Whenever $\tau^{d=0}\neq\tau^{d=1}$, Eq. (9) will be zero if and only if $\lambda_{0}=\lambda_{1}$. Conversely, if $\lambda_{0}\neq\lambda_{1}$, Eq. (9) will be zero if and only if $\tau^{d=0}=\tau^{d=1}$.

For a time-varying covariate, parallel trends as written in Eq. (8) requires either 1) zero mean difference between the groups at both times, $\tau_{t=1}^{d=1}-\tau_{t=1}^{d=0}=\tau_{t=0}^{d=1}-\tau_{t=0}^{d=0}=0$ (regardless of the effect of the covariate on the outcome at either time) or 2) a constant effect of the covariate on the outcome over time, $\lambda_{0}=\lambda_{1}$, *and* constant (non-zero) difference in the covariate means of the two groups, $\tau_{t=1}^{d=1}-\tau_{t=1}^{d=0}=\tau_{t=0}^{d=1}-\tau_{t=0}^{d=0}\neq0$.

# Appendix B: Adjusting for Time-Varying Covariates

In this section of the appendix, we discuss of the problems of adjusting for time-varying confounders as described in the “Adjusting for Time-Varying Confounders” sub-section of the introduction. A time-varying covariate that is *affected* by treatment and *affects* the outcome makes recovering the causal effect difficult. On one hand, failing to adjust for the time-varying covariate will result in failures of parallel trends. On the other hand, adjusting for the time-varying covariate, since it is on the pathway between treatment and the outcome, will adjust away some of the effect of treatment on the outcome, resulting in biased estimates.

We begin with notation that should be familiar to those who read our paper. $Y\left( t \right)$ is the continuous outcome measured at time $t$. For simplicity, we assume that$t \in\{0, 1\}$where $t = 0$ is the pre-treatment period and $t = 1$ the post-treatment period. Treatment is binary and represented by $D$. Finally, we have a time-varying covariate $X_{it}$ where $i$ in an index for a unit (e.g., a state or an individual). Let $\tau_{dt}=E\left[ X_{it} \mid D=d \right]$ be the covariate group-time mean. We also introduce counterfactual notation for the covariate so that $X_{it}^{d}$ is the (possibly counterfactual) value of $X$ for individual $i$and time $t$ under treatment $D = d$. Since we assume that treatment directly affects $X$, we may have that $X_{i1}^{0}\neq X_{i1}^{1}$.

Let's extend the notation for the covariate means to counterfactual world so that $E\left( X_{it}^{0} \mid D=d \right)=\tau_{dt}^{0}$ and $E\left( X_{it}^{1} \mid D=d \right)=\tau_{dt}^{1}$. We assume that treatment (which occurs between times 0 and 1) does not affect past versions of $X$ so that $\tau_{d0}^{0}=\tau_{d0}^{1}=\tau_{d0}$. We also assume that the covariate evolves differently in the two groups even absent treatment, leading to the failure of parallel trends. That is, $\tau_{01}-\tau_{00}\neq\tau_{11}-\tau_{10}$.

Suppose we have the same model for untreated outcomes as the main text:

$$E\left[ Y_{i}^{0}\left( t \right) | D=d_{i},X^{0}=x_{it}^{0} \right]=\alpha_{0}+\alpha_{1}d_{i}+\zeta_{t}+\lambda_{t}x_{it}^{0}.$$

For simplicity, let $\lambda_{t}= \lambda$. We can connect the untreated outcomes to the treated outcomes with a fixed treatment effect, $\gamma$: $Y_{i}^{1}\left( t \right)=Y_{i}^{0}\left( t \right)+ \gamma$.

Recall that the average treatment effect on the treated (ATT) is

$$ATT=E\left\{ Y^{1}\left( 1 \right)-Y^{0}\left( 1 \right) \mid D=1 \right\}.$$

Now, we have:

$$E\left\{ Y^{1}\left( 1 \right) \mid D=1 \right\}=\alpha_{0}+\alpha_{1}+\zeta_{1}+\lambda_{1}\tau_{11}^{1}+ \gamma$$

and

$$E\left\{ Y^{0}\left( 1 \right) \mid D=1 \right\}=\alpha_{0}+\alpha_{1}+\zeta_{1}+\lambda_{1}\tau_{11}^{0}.$$

Plugging into the ATT:

$$ATT=\lambda_{1}\tau_{11}^{1}+\gamma-\lambda_{1}\tau_{11}^{0}=\gamma+\lambda_{1}\left( \tau_{11}^{1}-\tau_{11}^{0} \right).$$

The ATT is what we want to calculate. Let’s look at estimates for the ATT from each an unadjusted model and a regression model that correctly adjusts for $X$.

**Unadjusted Estimator:**

$$\hat{ATT_{unadj}}=\left\{ E\left( Y\left( 1 \right) \mid D=1 \right)-E\left( Y\left( 0 \right) \mid D=1 \right) \right\}-$$

$$\left\{ E\left( Y\left( 1 \right) \mid D=0 \right)-E\left( Y\left( 0 \right) \mid D=0 \right) \right\}$$

$=\left\{ \alpha_{0}+\alpha_{1}+\zeta_{1}+\lambda_{1}\tau_{11}+\gamma-\left( \alpha_{0}+\alpha_{1}+\zeta_{0}+\lambda_{0}\tau_{10} \right) \right\}-$

$$\left\{ \alpha_{0}+\zeta_{1}+\lambda_{1}\tau_{01}-\left( \alpha_{0}+\zeta_{0}+\lambda_{0}\tau_{00} \right) \right\}$$

$$=\gamma+\zeta_{1}-\zeta_{0}+\lambda_{1}\tau_{11}-\lambda_{0}\tau_{10}-\left( \zeta_{1}-\zeta_{0}+\lambda_{1}\tau_{01}-\lambda_{0}\tau_{00} \right)$$

$$=\gamma+\lambda_{1}\tau_{11}-\lambda_{0}\tau_{10}-\lambda_{1}\tau_{01}+\lambda_{0}\tau_{00}=\gamma+\lambda_{1}\left( \tau_{11}-\tau_{01} \right)-\lambda_{0}\left( \tau_{10}-\tau_{00} \right).$$

Without significant restrictions on the $\lambda$ and $\tau$ values, this does not equal the ATT.

**Adjusted Estimator:**

Now, imagine we know which regression model to fit. In R, we can fit the model

lm(y ~ d*t + x*t), which is correctly specified. The estimate of the treatment effect will be the coefficient on the interaction between a (treatment indicator) and t (time). However, when we fit the model, we will get:

$$\hat{ATT_{adj}}= \gamma$$

which is biased for the true ATT.

# Appendix C: Calculation of ATT for Simulation Scenario 6

In the main paper, we state that the average treatment effect on the treated (ATT) in Scenario 6 is different than in the other scenarios. Here, we show our calculations for the ATT using our data-generating example. Below is the code used to generate data, using the dplyr R package.

dat <- expand.grid(id = 1:n, tp = 1:max.time) %>% arrange(id,tp) %>% group_by(id) %>%

mutate(int=rnorm(1,0,sd=0.25), # random intercept

p.trt=0.5, # probability of treatment

trt=rbinom(1, 1, p.trt), # treatment

x=rnorm(1, mean = 1.5 - 0.5*trt, sd = 1.5 - 0.5*trt),

post=I(tp >= trt.time), # indicator of post-treatment period

treated=I(post == 1 & trt == 1), # time-varying indicator if treated or not

x=ifelse(tp>=2, lag(x, 1) + (tp-1)/10 *

rnorm(1, mean = 1, sd = 0.1) - I(trt == 1) * I(tp>6)*(tp)/20, x)

) %>%

ungroup()

dat <- dat %>% mutate(err=rnorm(n*max.time),

y = 1 + x + trt + int + err + treated + ((tp - 2.5)^2)/10,

y.t = 1 + x * tp / 10 + trt +

int + err + treated + ((tp - 2.5)^2)/10) %>%

group_by(id) %>% mutate(y.diff = y - lag(y), y.diff2 = y.t - lag(y.t)) %>% ungroup()

To begin, we *only* need to look at the treated group since the ATT is defined on the treated population. The setup is relatively simple. We set $n = 1000$ to be the total number of units followed over 10 max.time time points. Units were assigned to the treatment group with probability 0.5. The treated units were given treatment beginning at $t = 6$; thus, we had five pre-treatment time points and five post-treatment time points. The covariate $X$ at baseline was drawn from a Normal distribution, $N\left( 1,1^{2} \right)$ from the treated population. During the pre-treatment period, the means of the covariate increased by about $\frac{1}{10}$ cumulatively from $t = 2, \ldots, 10$. However, the mean of the covariate was affected by treatment too, so that for the treated group when $t \geq6,$ the mean went down by an average of $\frac{1}{20}$ per time point.

| **Time** | $t = 1$ | $t = 2$ | $t = 3$ | $t = 4$ | $t = 5$ | $t = 6$ | $t = 7$ | $t = 8$ | $t = 9$ | $t = 10$ |
| --- | --- | --- | --- | --- | --- | --- | --- | --- | --- | --- |
| Mean($X^{0}$) | 1.0 | 1.1 | 1.2 | 1.3 | 1.4 | 1.5 | 1.6 | 1.7 | 1.8 | 1.9 |
| Mean($X^{1}$) | 1.0 | 1.1 | 1.2 | 1.3 | 1.4 | 1.45 | 1.5 | 1.55 | 1.6 | 1.65 |

Table: Evolution of counterfactual means of covariate X for the treated group.

Note that for this simulation scenario, we have two different outcomes. In the first, denoted y, the effect of $X$ on the outcome is the same at every time point. For the second outcome, denoted

y.t, the covariate has a time-varying effect on the outcome. The two outcome processes are detailed below:

$$y=1+x+trt+int+err+treated+\left( \left( tp-2.5 \right)^{2} \right)/10$$

$$y.t = 1 + x * tp / 10 + trt + int + err + treated + ((tp - 2.5)^2)/10).$$

So this difference is that in the second equation, $X$ interacts with time. Note that both $int$ and $err$ are mean zero normal random variables and $treated = 1$ whenever $tp >5$. (We are only

considering the treated group. This would not be true for the comparison group.) Like we did for the mean of $X$, we can build a table for the means of $Y$ using the above equations.

For y, we get the following results:

| **Time** | $t = 1$ | $t = 2$ | $t = 3$ | $t = 4$ | $t = 5$ | $t = 6$ | $t = 7$ | $t = 8$ | $t = 9$ | $t = 10$ | Avg pre | Avg post |
| --- | --- | --- | --- | --- | --- | --- | --- | --- | --- | --- | --- | --- |
| Mean($Y^{0}$) | 3.225 | 3.125 | 3.225 | 3.525 | 4.025 | 4.725 | 5.625 | 6.725 | 8.025 | 9.525 | 3.425 | 6.925 |
| Mean($Y^{1}$) | 3.225 | 3.125 | 3.225 | 3.525 | 4.025 | 5.675 | 6.525 | 7.575 | 8.825 | 10.275 | 3.425 | 7.775 |

We'll calculate a few of these by hand to give an idea of what we're doing. Take the mean of $Y^{0}$ at $t = 7$:

$$y=1+x+trt+int+err+treated+\left( \left( tp-2.5 \right)^{2} \right)/10$$

$$=1+x+1+0+0+0+\left( 7-2.5 \right)^{2}/10$$

$$=1+1.6+1+0+0+0+\left( 7-2.5 \right)^{2}/10$$

$$= 5.625.$$

Here, we plugged in 1.6 for $x$ since it equals the untreated mean of the covariate (see Table D1). Both $int$ and $err$ are independent mean zero random variables so we plug in 0.

Following similar calculations, the mean of $Y^{1}$ at $t = 7$ is:

$$y=1+x+trt+int+err+treated+\left( \left( tp-2.5 \right)^{2} \right)/10$$

$$=1+x+1+0+0+1+\left( 7-2.5 \right)^{2}/10$$

$$=1+1.5+1+0+0+1+\left( 7-2.5 \right)^{2}/10$$

$$= 6.525.$$

The ATT here is $7.775 - 6.925 = 0.85$, which is calculated by taking the mean of the last 5 columns (the post-treatment time points) for each row and subtracting them.

And for y.t, we get the following results:

| **Time** | $t = 1$ | $t = 2$ | $t = 3$ | $t = 4$ | $t = 5$ | $t = 6$ | $t = 7$ | $t = 8$ | $t = 9$ | $t = 10$ | Avg pre | Avg post |
| --- | --- | --- | --- | --- | --- | --- | --- | --- | --- | --- | --- | --- |
| Mean($Y^{0}$) | 2.325 | 2.245 | 2.385 | 2.745 | 3.325 | 4.125 | 5.145 | 6.385 | 7.845 | 9.525 | 2.605 | 6.605 |
| Mean($Y^{1}$) | 2.325 | 2.245 | 2.385 | 2.745 | 3.325 | 5.095 | 6.075 | 7.265 | 8.665 | 10.275 | 2.605 | 7.475 |

Table: Evolution of counterfactual means of outcome Y for the treated group.

The ATT here equals 0.87.

# Appendix D: Data-Generating Distributions for Simulations

**Table D1**: Data-generating models for simulations with a time-invariant covariate

| Scenario | Data-Generating Model |
| --- | --- |
| 1: Time-invariant covariate effect | $X_{i} \sim N\left( m\left( d_{i} \right),v\left( d_{i} \right) \right)$ $Y_{i}\left( t \right) \sim N\left( 1+d_{i}+d_{i}p_{t}+u_{i}+x_{i}+f\left( t \right),1 \right)$ |
| 2: Time-varying covariate effect | $X_{i} \sim N\left( m\left( d_{i} \right), v\left( d_{i} \right) \right)$ $Y_{i}\left( t \right) \sim N\left( 1+d_{i}+d_{i}p_{t}+u_{i}+x_{i}+f\left( t \right)+g\left( x_{i},t \right),1 \right)$ |
| 3: Treatment-independent covariate | $X_{i} \sim N\left( 1,1 \right)$ $Y_{i}\left( t \right) \sim N\left( 1+d_{i}+d_{i}p_{t}+u_{i}+x_{i}+f\left( t \right)+g\left( x_{i},t \right),1 \right)$ |

**Legend**: $Y_{i}\left( t \right)$: outcome for $i^{th}$ subject at time $t$, $x_{i}$: covariate, $d_{i}$: group indicator, $u_{i}$: random intercept, $p_{t}$: indicator of post-treatment time point. The treatment assignment for all scenarios is $d_{i} \sim\text{Bernoulli}\left( 0.5 \right)$ and the unit-level intercepts are $u_{i}\sim N\left( 0,.{25}^{2} \right)$. Lastly, $m\left( d_{i} \right)=1.5-0.5d_{i}$, $f\left( t \right)=\left( t-2.5 \right)^{2}/10$, $g\left( x_{i},t \right)=\left( x_{i}\cdot t \right)/10$, and $v\left( d_{i} \right)=\left( 1.5-0.5d_{i} \right)^{2}$.

**Table D2**: Data-generating models for simulations with a time-invariant covariate

| Scenario | Data-Generating Model |
| --- | --- |
| 4: Parallel evolution | $X_{ti}=x_{\left( t-1 \right)i}+m_{1}\left( t \right)\cdot z$ $Y_{i}\left( t \right) \sim N\left( 1+d_{i}+d_{i}p_{t}+u_{i}+x_{ti}+f\left( t \right)+g\left( x_{i},t \right),1 \right)$ |
| 5: Evolution differs by group | $X_{ti}=x_{\left( t-1 \right)i}+m_{2}\left( d_{i},t \right)\cdot z$  $Y_{i}\left( t \right) \sim N\left( 1+d_{i}+d_{i}p_{t}+u_{i}+x_{ti}+f\left( t \right)+g\left( x_{i},t \right),1 \right)$ |
| 6: Evolution diverges in post-treatment period | $X_{ti}=x_{\left( t-1 \right)i}+m_{1}\left( t \right)\cdot z-m_{3}\left( d_{i},t \right)$  $Y_{i}\left( t \right) \sim N\left( 1+d_{i}+d_{i}p_{t}+u_{i}+x_{ti}+f\left( t \right)+g\left( x_{i},t \right),1 \right)$ |

**Legend:** $Y_{i}\left( t \right)$: outcome for $i^{th}$ subject at time $t,$ $x_{i}$: covariate, $d_{i}$: group indicator, $u_{i}$: random intercept, $p_{t}$: indicator of post-treatment time point. The treatment assignment for all scenarios is $d_{i} \sim\text{Bernoulli}\left( 0.5 \right)$. The unit-level intercepts are $u_{i} \sim N\left( 0,.{25}^{2} \right)$. The covariate value at the first time point is $X_{1i} \sim N\left( 1.5-0.5d_{i},\left( 1.5-0.5d_{i} \right)^{2} \right)$. And $z\sim N\left( {1,0.1}^{2} \right)$. The functions that govern the evolution of the covariate are $m_{1}\left( t \right)=\left( t-1 \right)/10$, $m_{2}\left( d_{i},t \right)=\left( I_{d_{i}=1}-I_{d_{i}=0} \right)\left( t-1 \right)/10$, and $m_{3}\left( d_{i},t \right)=d_{i}p_{t}t/20$. Lastly, the outcome process (a) refers to those where the covariate has a time-invariant effect on the outcome and uses the functions $f\left( t \right)=\left( t-2.5 \right)^{2}/10$and $g\left( x_{i},t \right)=0$. The outcome process (b) refers to the simulations where the covariate has a time-varying effect on the outcome and uses the functions $f\left( t \right)=\left( t-2.5 \right)^{2}/10$ and $g\left( x_{i},t \right)=\left( x_{i}\cdot t \right)/10$.
